# Incidence of Japanese Encephalitis among Acute Encephalitis Syndrome Cases in West Bengal, India

**DOI:** 10.1155/2013/896749

**Published:** 2013-11-11

**Authors:** Bhaswati Bandyopadhyay, Indrani Bhattacharyya, Srima Adhikary, Saiantani Mondal, Jayashree Konar, Nidhi Dawar, Asit Biswas, Nemai Bhattacharya

**Affiliations:** ^1^Calcutta School of Tropical Medicine, 108 C.R. Avenue, Kolkata 700073, India; ^2^NRHM, Government of West Bengal, Swasthya Bhavan Sector V, Salt Lake, Kolkata 700091, India

## Abstract

*Background and Objectives*. Japanese encephalitis (JE) is the most important cause of acute and epidemic viral encephalitis. Every year sporadic JE cases are reported from the various districts of West Bengal, indicating its endemicity in this state. JE vaccination programme has been undertaken by the State Health Department of West Bengal. This study was aimed at seeing the present scenario of JE among acute encephalitis syndrome (AES) cases in West Bengal. *Materials and Methods*. Blood and/or CSF samples were referred from suspected AES cases to the referral virology laboratory of the Calcutta School of Tropical Medicine from different hospitals of Kolkata. IgM antibody capture ELISA was performed on the CSF and serum samples by JE virus MAC ELISA kit supplied by the National Institute of Virology, Pune. *Results*. The present study reveals that 22.76% and 5% of the AES cases were positive for JE IgM in 2011 and 2012, respectively. JE is mainly prevalent in children and adolescents below 20 years of age with no gender predilection. Although the percentages of JE positive cases were high in 2011, it sharply decreased thereafter possibly due to better awareness programs, due to mass vaccination, or simply due to natural epidemiological niche periodicity due to herd immunity.

## 1. Introduction

The mosquito-borne Japanese encephalitis virus (JEV) is an enveloped, positive-sense single-stranded RNA virus and member of the genus *Flavivirus* under the family Flaviviridae [1]. JEV is the sole etiologic agent of Japanese Encephalitis (JE), a neurotropic killer disease being one of the major causes of viral encephalitis in human. Since the isolation of this virus in Japan in 1935 [2], it has spread worldwide becoming a major public health problem. JE is a disease of major public health importance due to its high epidemic potential, high case fatality rate (CFR), and sequelae among survivors [3].

Approximately 2 billion people live in countries where JE presents a significant risk to humans and animals, particularly in China and India, with at least 700 million potentially susceptible children [4]. In Southeast Asia around 50,000 cases and 10,000 deaths occur per year affecting essentially children below 10 years of age [5]. Further threats to humanity are there because the JE virus has shown a tendency to extend to other geographic areas. The combined effects of climate change, altered bird migratory patterns, increasing movement of humans, animals, and goods, increasing deforestation, and development of irrigation projects will also help this geographic dispersal of the virus, producing an enhanced threat. The disease is also highly prevalent in animals. In Nepal, seroprevalence of JE in pigs, ducks, and horses was 48.11%, 26.79%, and 50.0%, respectively [6]. Phylogenetic analysis showed that JE isolates in India belonged to genogroup III [7].

Although most human infections are mild or asymptomatic, about 50% of patients who develop encephalitis suffer permanent neurologic defects and 30% of them die due to the disease [8]. In 1973, JE outbreak was first recorded in the districts of Burdwan and Bankura in West Bengal where 700 cases and 300 deaths were reported [9–13].

Since 1973, epidemics of JE have occurred in West Bengal, Bihar, Uttar Pradesh, Assam, Andhra Pradesh, Tamil Nadu, and Karnataka [14]. Every year sporadic JE cases are reported indicating their endemicity in this state [15]. JE vaccination programme has been undertaken by the State Health Department, Government of West Bengal.

This study was aimed to see the present scenario of JE among acute encephalitis syndrome cases in West Bengal.

## 2. Materials and Methods

### 2.1. Human Blood and or CSF Samples

Blood and/or CSF samples were referred and submitted to the referral Virology laboratory at the Calcutta School of Tropical Medicine, from 606 clinically diagnosed cases of acute encephalitis syndrome during the period from January 2011 to December 2012. Specimen collection and transportation of samples were strictly monitored. 1 mL CSF and 2–5 mL of clotted blood sample were collected as per standard procedures. The samples were transported to the virology laboratory maintaining cold chain. The CSF and serum samples were stored at 4°C in the refrigerator if tested within 3 days or minus 80 degree freezer for long-term storage.

### 2.2. Serological Study for JE

IgM antibody capture (MAC) ELISA was performed on the CSF and serum samples by JE virus MAC ELISA kit supplied by the National Institute of Virology, Pune, as an integral part of the National Vector Borne Disease Control Program. The samples were tested strictly following the manufacturer's protocol.

## 3. Results

The present study was carried out in the Virology unit of the Microbiology Department of the Calcutta School of Tropical Medicine, Kolkata, and comprised 606 clinically diagnosed cases of acute encephalitic syndrome. Among them 357 (59.92%) were males and 249 (41.08%) were females. 74 (12.21%) cases were found to be positive for JE.  Table 1 shows that 23.84% and 21.05% of the JE positive cases were males and females, respectively, in 2011, whereas 4.8% and 5.2% of the JE positive cases were males and females, respectively, in 2012. In general, the differences between male and female distributions of JE positive cases were not statistically significant at a 95% level.

However, there was a remarkable change in the percentages of JE positive cases in the years 2011 and 2012 (Table 1). In males, the percentage of JE positive cases dropped from 23.84% to only 4.8% (*P* value is highly significant below 0.01 level); similarly in females it dropped from 21.05% to 5.2% (*P* value is highly significant below 0.01 level).

**Figure 1:**
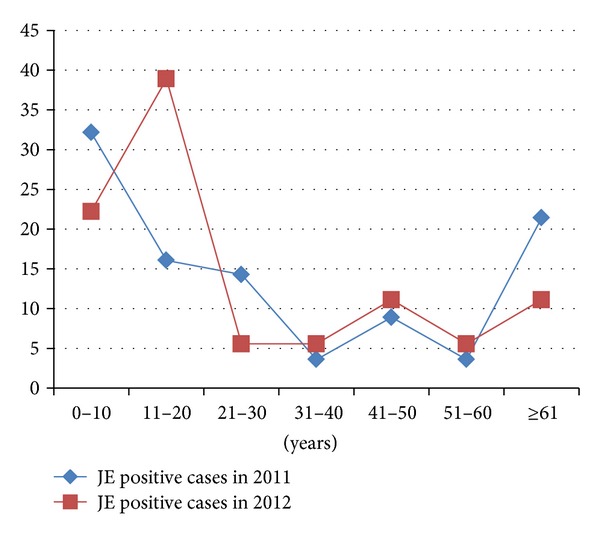
Percentage of JE positive cases in the various age groups, 2011-2012.

Figure 1 shows the distribution of the percentage of JE positive cases in the different age groups in the years 2011 and 2012. It was found that 48.21% and 61.11% of JE positive cases were below 20 years of age in 2011 and 2012, respectively.

**Figure 2:**
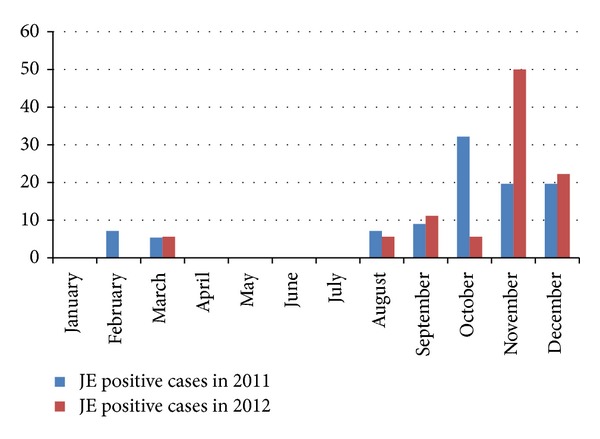
Monthly distribution of JE positive cases (in percentage), 2011-2012.

Figure 2 shows the monthly distribution of the JE positive cases (in percentage). It is evident that a larger number of JE cases occurred in the rainy season and after the rainy season.

**Figure 3:**
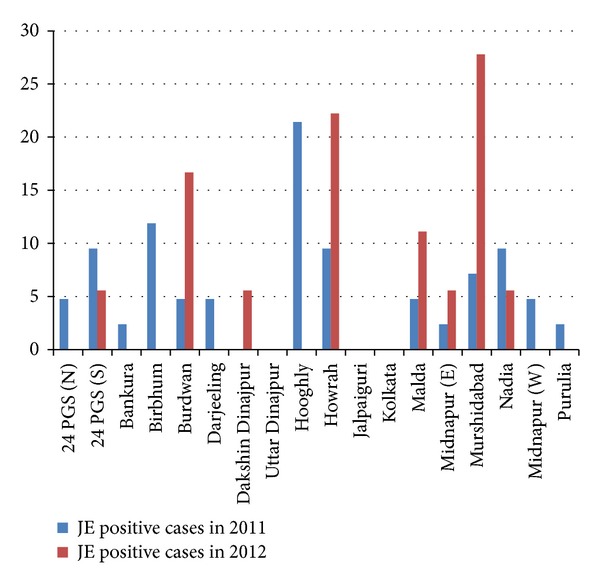
District wise distribution of JE positive cases (in percentage), 2011-2012.

Figure 3 shows that sporadic JE positive cases were reported from almost all rural districts of West Bengal. Maximum number of JE IgM positive cases occurred in Hooghly district followed by Birbhum in 2011. However, comparatively a larger number of cases were reported from Murshidabad, Bardhaman, and Howrah districts of West Bengal in 2012.

Out of 56 JE cases in 2011 line listing could be done in 42 cases. No address was available for 14 patients in 2011 as these cases were referred from other Medical colleges of West Bengal. 

## 4. Discussion

Patients with high grade fever (≥39°C) for 5–15 days including any 2 of the following symptoms, namely, headache, vomiting, stupor, delirium, abnormal movements, presence of kernig's sign, convulsions, neck rigidity, altered sensorium, and unconsciousness were considered as acute encephalitis syndrome (AES) cases [16]. Although these manifestations can occur in manifold infectious diseases, in West Bengal, JE is an important disease particularly in rural areas and should be considered first in such cases. In general population the incidence of acute encephalitis syndrome ranges between 3.5 and 7.4 cases per 100,000 patient-years [17].

The incidence of JE varied in each month in our study. However, the most of the cases were reported during the monsoon and after the monsoon period. No patients were admitted during April to July. Anuradha et al. [3], Sarkar et al. [18], Benakappa et al. [19], and Reuben and Gajanana [20] have also reported higher incidence of JE during similar months due to increased prevalence of the vector mosquitoes. *Culex *mosquitoes breed abundantly in the paddy fields covered with stagnant water during the rainy season. Most of the JE cases occurred in children and adolescents below 20 years of age. Children and adolescents are probably directly exposed to the mosquito vector (*Culex *sp.) bite, as they often visit the fields with their parents or may take active part in the cultivation where vectors are abundant. Also lack of immunity against JE virus in the younger age group could be responsible for the increased incidence of disease in this age group [21–23].

Our study also indicates that most of the JE cases occurred in the rural districts of West Bengal, where the main occupation is farming. This finding is also similar to the findings of Anuradha et al. [3], Benakappa et al. [19], and Reuben and Gajanana [20].

There are currently believed to be four distinct genotypes of JEV, genotypes I to IV [24], although some studies support the existence of a fifth JEV genotype [25, 26], all of which are thought to have arisen from a common ancestor virus present in the Indonesian-Malaysian region [24]. While some genotypes are present in multiple countries (such as genotype III), others are present in only one country, such as genotype IV which is found only in Indonesia [24]. Conversely, countries may experience the circulation of several genotypes, such as Indonesia where genotypes II, III, and IV circulate [24]. However, genotypic shift, with the replacement of one genotype by another, has occurred recently in several countries [27–29]. Currently, JEV is considered hyperendemic in northern India and southern Nepal as well as in parts of central and southern India. More than 3 billion people are living in the current JE-endemic region [30, 31].

However, during the past decade, JEV GI has been introduced into Republic of Korea, Thailand, and China and has replaced the GIII strains that had been circulating in Japan and Vietnam during the mid 1990s [32]. Until 2007, all known JEV strains isolated in India belonged to GIII [31, 33–35].

Sarkar et al. reported the prevalence of genotypes III and I among the JE cases of West Bengal [18]. Studies from Gorakhpur also indicate the presence of genotypes I and III isolates among the AES cases [36].

The present study reveals that 22.76% and 5% of the AES cases were positive for JE IgM in 2011 and 2012, respectively. There was no sex predilection among the JE cases in the population of West Bengal, India. Results from a previous study in 2010 done by Sarkar et al. [18] on JE seropositivity in West Bengal and a similar study done in Uttar Pradesh of India in 2011 by Bhatt et al. [37] were comparable to our findings of 2011. Thus, it appears that although the percentages of JE positive cases were more or less stable in 2010 and in 2011, after 2011 it decreased sharply. This may be due to better awareness programs, mass vaccination, and/or simply due to natural epidemiological niche periodicity due to herd immunity. A changing epidemiological trend of flavivirus mediated diseases from JE to dengue has also been noted in recent years possibly due to increased urbanisation of the remote villages [38–40]. Cross-protection by other flaviviral diseases, namely, dengue, could be a reason for decline of the JE cases to some extent. The State Health Department of Government of West Bengal undertook mass vaccination programme against JE in several endemic districts using live attenuated JE vaccine SA-14-14-2. The significant decline of JE cases in our study in 2012 could be attributed to this as a major factor for the controlling of JE cases in the previously endemic district. However, active surveillance of JE cases is still warranted in order to be vigilant about any new genotype introduction in the endemic districts or to find out any spread into newer geographical areas. 

## Figures and Tables

**Table 1:** Distribution of JE positive cases in 2011 and 2012.

Sex	Samples tested in 2011	Samples reactive in 2011	Samples tested in 2012	Samples reactive in 2012
Male	151	36 (23.84%)	206	10 (4.8%)
Female	95	20 (21.05%)	154	8 (5.2%)

Total	246	56 (22.76%)	360	18 (5%)

## References

[1] Lindenbach BD, Rice CM, Knipe DM, Howley PM (2001). Flaviviridae: the viruses and their replication. *Field’s Virology*.

[2] Tanaka M, Aira Y, Igarashi A (1991). Comparative nucleotide and amino acid sequences of five Japanese encephalitis virus strains isolated in Japan and China. *Tropical Medicine*.

[3] Anuradha SK, Surekha YA, Sathyanarayan MS (2011). Epidemiological aspects of Japanese encephalitis in Bellary, Karnataka, India. *International Journal of Biological and Medical Research*.

[4] Gould EA, Solomon T, Mackenzie JS (2008). Does antiviral therapy have a role in the control of Japanese encephalitis?. *Antiviral Research*.

[5] Diagana M, Preux P-M, Dumas M (2007). Japanese encephalitis revisited. *Journal of the Neurological Sciences*.

[6] Pant GR (2006). A serological survey of pigs, horses, and ducks in Nepal for evidence of infection with Japanese encephalitis virus. *Annals of the New York Academy of Sciences*.

[7] Parida M, Dash PK, Tripathi NK (2006). Japanese encephalitis Outbreak, India, 2005. *Emerging Infectious Diseases*.

[8] Babu GN, Kalita J, Misra UK (2006). Inflammatory markers in the patients of Japanese encephalitis. *Neurological Research*.

[9] Ghosh SN, Rodrigues FM, Seth GP (1975). Investigations on the outbreak of Japanese encephalitis in Burdwan district, west Bengal. Part II. Serological survey of human population. *Indian Journal of Medical Research*.

[10] Rodrigues FM, Ghosh SN, Banerjee K (1975). A post epidemic serological survey of humans in Bankura district, west Bengal, following the epidemic of Japanese encephalitis in 1973. *Indian Journal of Medical Research*.

[11] Rajagopalan PK, Panicker KN (1978). A note on the 1976 epidemic of Japanese encephalitis in Burdwan district, west Bengal. *The Indian Journal of Medical Research*.

[12] Banerjee K, Sengupta SN, Dandawate CN (1976). Virological and serological investigations of an epidemic of encephalitis which occurred at Bankura district, west Bengal. *Indian Journal of Medical Research*.

[13] Mukhopadhyay BB, Mukherjee B, Bagchi SB, Chakraborty M, Mukherjee KK, Mukherjee MK (1990). An epidemiological investigation of Japanese encephalitis outbreak in Burdwan, district of west Bengal during 1987–1988. *Indian Journal of Public Health*.

[14] Mohan Rao CV, Prasad SR, Rodrigues JJ, Sharma NG, Shaikh BH, Pavri KM (1983). The first laboratory proven outbreak of Japanese encephalitis in Goa. *The Indian Journal of Medical Research*.

[15] Sarkar A, Taraphdar D, Mukhopadhyay SK, Chakrabarti S, Chatterjee S (2012). Serological and molecular diagnosis of Japanese encephalitis reveals an increasing public health problem in the state of west Bengal, India. *Transactions of the Royal Society of Tropical Medicine and Hygiene*.

[16] Solomon T, Thi TT, Lewthwaite P (2008). A cohort study to assess the new WHO Japanese encephalitis surveillance standards. *Bulletin of the World Health Organization*.

[17] Granerod J, Crowcroft NS (2007). The epidemiology of acute encephalitis. *Neuropsychological Rehabilitation*.

[18] Sarkar A, Taraphdar D, Mukhopadhyay SK, Chakrabarti S, Chatterjee S (2012). Molecular evidence for the occurrence of Japanese encephalitis virus genotype I and III infection associated with acute encephalitis in patients of west Bengal, India, 2010. *Virology Journal*.

[19] Benakappa DG, Anvekar GA, Viswanath D, George S (1984). Japanese encephalitis. *Indian Pediatrics*.

[20] Reuben R, Gajanana A (1997). Japanese encephalitis in India. *Indian Journal of Pediatrics*.

[21] Burke DS, Lorsomrudee W, Leake CJ (1985). Fatal outcome in Japanese encephalitis. *American Journal of Tropical Medicine and Hygiene*.

[22] Libraty DH, Nisalak A, Endy TP, Suntayakorn S, Vaughn DW, Innis BL (2002). Clinical and immunological risk factors for severe disease in Japanese encephalitis. *Transactions of the Royal Society of Tropical Medicine and Hygiene*.

[23] Halstead SB, Jacobson J (2003). Japanese encephalitis. *Advances in Virus Research*.

[24] Solomon T, Ni H, Beasley DWC, Ekkelenkamp M, Cardosa MJ, Barrett ADT (2003). Origin and evolution of Japanese encephalitis virus in southeast Asia. *Journal of Virology*.

[25] Li MH, Fu SH, Chen WX (2011). Genotype v Japanese encephalitis virus is emerging. *PLoS Neglected Tropical Diseases*.

[26] Mohammed MAF, Galbraith SE, Radford AD (2011). Molecular phylogenetic and evolutionary analyses of Muar strain of Japanese encephalitis virus reveal it is the missing fifth genotype. *Infection, Genetics and Evolution*.

[27] Ma S-P, Yoshida Y, Makino Y, Tadano M, Ono T, Ogawa M (2003). Short report: a major genotype of Japanese encephalitis virus currently circulating in Japan. *The American Journal of Tropical Medicine and Hygiene*.

[28] Nga PT, del Carmen Parquet M, Cuong VD (2004). Shift in Japanese Encephalitis Virus (JEV) genotype circulating in northern Vietnam: implications for frequent introductions of JEV from southeast Asia to east Asia. *Journal of General Virology*.

[29] Yoshida Y, Tabei Y, Hasegawa M, Nagashima M, Morozumi S (2005). Genotypic analysis of Japanese encephalitis virus strains isolated from Swine in Tokyo, Japan. *Japanese Journal of Infectious Diseases*.

[30] Misra UK, Kalita J (2010). Overview: Japanese encephalitis. *Progress in Neurobiology*.

[31] Mackenzie JS, Gubler DJ, Petersen LR (2004). Emerging flaviviruses: the spread and resurgence of Japanese encephalitis, west Nile and dengue viruses. *Nature Medicine*.

[32] Huang JH, Lin TH, Teng HJ (2010). Molecular epidemiology of Japanese encephalitis virus, Taiwan. *Emerging Infectious Diseases*.

[33] Saxena SK, Mishra N, Saxena R, Singh M, Mathur A (2009). Trend of Japanese encephalitis in north India: evidence from thirty-eight acute encephalitis cases and appraisal of niceties. *Journal of Infection in Developing Countries*.

[34] Sapkal GN, Bondre VP, Fulmali PV (2009). Enteroviruses in patients with acute encephalitis, Uttar Pradesh, India. *Emerging Infectious Diseases*.

[35] Uchil PD, Satchidanandam V (2001). Phylogenetic analysis of Japanese encephalitis virus: envelope gene based analysis reveals a fifth genotype, geographic clustering, and multiple introductions of the virus into the Indian subcontinent. *American Journal of Tropical Medicine and Hygiene*.

[36] Fulmali PV, Sapkal GN, Athawale S, Gore MM, Mishra AC, Bondre VP (2011). Introduction of Japanese encephalitis virus genotypei, India. *Emerging Infectious Diseases*.

[37] Bhatt GC, Bondre VP, Sapkal GN (2012). Changing clinico-laboratory profile of encephalitis patients in the eastern Uttar Pradesh region of India. *Tropical Doctor*.

[38] Bandyopadhyay B, Bhattacharyya I, Adhikary S (2013). A comprehensive study on the 2012 Dengue fever outbreak in Kolkata, India. *ISRN Virology*.

[39] Taraphdar D, Sarkar A, Bhattacharya MK, Chatterjee S (2010). Sero diagnosis of dengue activity in an unknown febrile outbreak at the Siliguri Town, districtrict Darjeeling, west Bengal. *Asian Pacific Journal of Tropical Medicine*.

[40] Sarkar A, Taraphdar D, Chatterjee S (2010). Investigations of recurrent out breaks of unknown fever, establish rural dengue activity in west Midnapore, a costal district in west Bengal, India. *Archives of Clinical Microbiology*.

